# Extrinsic Calibration between a Camera and a 2D Laser Rangefinder using a Photogrammetric Control Field

**DOI:** 10.3390/s19092030

**Published:** 2019-04-30

**Authors:** Jia Fan, Yuchun Huang, Jie Shan, Shun Zhang, Fei Zhu

**Affiliations:** 1 School of Remote Sensing and Information Engineering, Wuhan University, Wuhan 430079, China; fanjia@whu.edu.cn (J.F.); shun_z@whu.edu.cn (S.Z.); f_zhuwhu@whu.edu.cn (F.Z.); 2 Lyles School of Civil Engineering, Purdue University, West Lafayette, IN 47907, USA; jshan@purdue.edu

**Keywords:** 2D laser rangefinder, camera, extrinsic calibration, photogrammetric control field, direct linear transform, perspective-three-point

## Abstract

The combination of a camera and a 2D laser rangefinder (LRF) is widely used in robotics, mapping, and unmanned driving to simultaneously obtain the 3D geometry and color texture of a scene. However, data misregistration between the camera and the LRF frequently occurs due to the difficulty of precise installation and alignment between them. Extrinsic calibration between the camera and the LRF is necessary. In this study, a photogrammetric control field is used to perform a robust and accurate calibration between the camera and the LRF which requires only one shot. With the use of the photogrammetric control field as the common reference, extrinsic calibration between two sensors is decoupled by calibrating each sensor separately. First, using the room corner of the control field, the LRF is calibrated with respect to the control field by solving a simplified perspective-three-point problem. Then, a large number of control points are used to obtain the robust and accurate extrinsic parameters of the camera with respect to the control field. Experiments with simulated and real data were performed. The experimental results show that the proposed scheme is accurate, precise, and robust under different noise levels, and the calibration results can be utilized in indoor and outdoor applications.

## 1. Introduction

High-resolution cameras and 2D laser rangefinders (LRFs) are often combined in mobile mapping [1], object detection [2], and simultaneous localization and mapping [3,4] due to their small size, low cost, and high flexibility. The camera can provide high-resolution color and texture information of the surrounding scene, while the LRF can collect high-precision distance information. To take full advantages of the two sensors and obtain the 3D geometry and color texture information of a scene, data fusion of the two sensors is needed. However, data misregistration of the camera and LRF often occurs due to the difficulty of precise installation and alignment between them. To address this problem, the extrinsic parameters between the two sensors, i.e., the rotation and translation between the camera and LRF coordinate systems, need to be calibrated.

The extrinsic calibration between multiple sensors is normally accomplished through corresponding features of the data captured by the sensors. However, it is difficult to accurately locate the corresponding points or other features between the image and the laser range data due to the following reasons. First, the laser range data captured by the 2D LRF record only one line formed by the intersection points of the laser scanning plane and the object surface. Unlike the 3D point clouds and the image in which we can obtain many features like corners and edges, no such feature is obtained in the line recorded by the LRF. Second, the laser range data are invisible in the image since the laser used by the LRF is outside the visible spectrum. Therefore, finding an effective approach for accurate extrinsic calibration between the 2D LRF and the camera is necessary and challenging.

Various calibration methods have been reported, most of which used the chessboard as the calibration pattern. The relative position of the camera with respect to the chessboard can be determined with the grid points of the chessboard. Besides, it is easy to recognize the laser points on the chessboard since it is a plane. Thus, the constraints between the camera and the LRF are established with the chessboard. Zhang and Pless [5] used a chessboard to establish a rigorous calibration method with the point-on-plane constraint which means that the laser points should be on the chessboard plane. This approach requires at least five shots of different poses to obtain an initial closed-form solution for the extrinsic parameters. More than 20 shots are often required to acquire robust initials, which makes the calibration time consuming. Moreover, point-on-plane constraints of all the laser points were used to perform the optimization to improve the initials. The optimized solution may converge to a local minimum due to the unstable initials. Based on Zhang’s method, Kassir and Peynot [6] proposed a reliable automatic camera-laser calibration toolbox. Zhou and Deng [7] used line-on-plane constraints, that is, the laser direction vector is perpendicular to the chessboard normal vector, to decouple the rotation matrix from the translation vector, i.e., to resolve the rotation and translation separately. The same constraints were used to optimize the initial values. This method theoretically required three different shots to solve the initial extrinsic parameters and more shots to perform the non-linear optimization. Vasconcelos et al. [8] used a chessboard as the calibration pattern to transform the point-line-plane constraints in 3D space into a perspective-three-point (P3P) problem. After getting the initial extrinsic parameters, the optimization was performed by minimizing the laser projection error. However, it required three shots to solve the initials, and suffered from multiple solutions and degeneration problems in solving P3P [9,10,11].

In addition, several methods used boards with specially designed shapes including the triangle, V-shaped, and cubic boards, which were composed of one plane, two planes, and three planes, respectively, as the calibration pattern. Li et al. [12] used a triangle board as the calibration pattern to capture the point-on-line constraints, that is, the projection of laser points lie on the corresponding lines in the image, between the laser points and triangular sides. Wasielewski and Strauss [13] used a V-shaped board to extract the intersections in laser points and the intersection lines of two planes in the image and optimized the extrinsic parameters using the point-on-line constraint. Sim et al. [14] refined the constraints by using three point-on-line constraints of a V-shaped board. Although these methods improved the constraints between the two sensors, they still required multiple shots at the target in different poses and relied on good initial estimation of extrinsic parameters. Recently, several scholars have presented improved calibration methods based on a trihedron. Chen et al. [15] used a cubic pattern and the point-on-line constraint for the extrinsic calibration of a 2D LRF and a camera, which required at least three shots of the target. Gomez-Ojeda et al. [16] introduced a method to calibrate a 2D LRF and a camera by observing the corners in human-made scenes. This method used line-on-plane and point-on-plane constraints to build the relationship between the two sensors to solve the initial values and optimize the result. However, this method requires three shots to obtain the initial extrinsic parameters and more shots to ensure an accurate result. 

The abovementioned methods have two major limitations. First, although they take multiple shots, some ill-posed shots may mislead the solution and cause the solution to become unstable. Second, they suffer from multiple solutions or early converge to a local minimum, which may lead to incorrect calibration results. Hu et al. [17] presented an extrinsic calibration method that only requires one shot at the target and obtained a unique solution. They used a trirectangular trihedron as the calibration pattern and estimated the poses of the camera and LRF using perspective-three-line [18,19] and P3P, respectively. For the LRF calibration, they formed a simplified P3P problem using the trirectangular trihedron and avoided multiple solutions and degeneration problems of P3P. For the camera calibration, they used the length of two edges to derive the real solution. However, accurate measurement of the length of edges could be difficult. In addition, the length of the edges was determined based on two endpoints. The lengths of the edges are inaccurate when the endpoints are noisy. Thus, they are sensitive to noise. 

**Figure 1:**
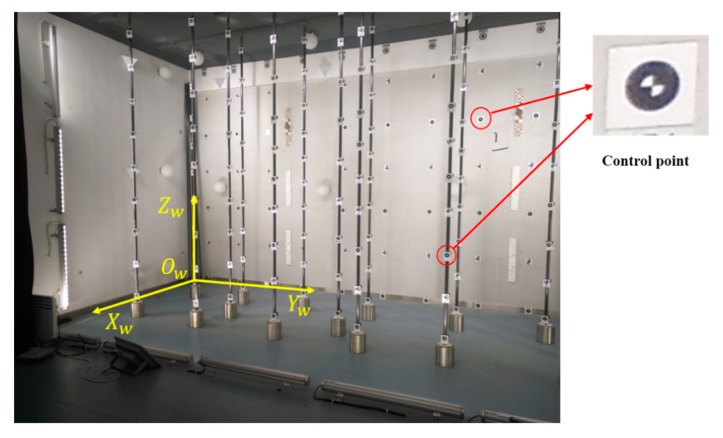
Photogrammetric control field.

This paper presents a scheme to calibrate the extrinsic parameters between the camera and 2D LRF using a photogrammetric control field to address the above limitations. The proposed scheme provides accurate and robust calibration results, and requires only one shot at the control field. A photogrammetric control field is frequently used in the calibration of the camera [20,21] and 3D laser scanner [22]. It brings no extra complexity to use the existing control field for extrinsic calibration between the camera and 2D LRF. A typical photogrammetric control field is shown in Figure 1. It is composed of a number of control points evenly distributed in a 3D space. The coordinates of the control points in the control field are accurately measured in advance. The coordinates of the control points in the image can be accurately obtained due to the distinct crossing of black and white sectors at the center, as shown in Figure 1. This study utilizes the control field as a common reference to calibrate the two sensors separately and successfully decouples the calibration process. 

Extrinsic calibration between the camera and the LRF was divided into LRF calibration with respect to the control field and camera calibration with respect to the control field. The P3P and direct linear transformation (DLT) [23,24] were employed to obtain the extrinsic parameters of the two sensors, respectively, with respect to the control field. The main contributions of this paper are summarized as follows:

1. The proposed calibration scheme required only one shot at the control field to accurately calibrate the extrinsic parameters. Compared with the calibration methods which needed multiple shots, it makes data collection easier.
2. The proposed calibration scheme is robust. The use of an elaborately designed control field not only avoids degeneration problems in the camera calibration, but also provides redundant observations to enhance its robustness. In addition, the use of the control field avoids degeneration problems in LRF calibration and provides a unique solution to traditional P3P problems by using a 3D right triangle pyramid formed by the LRF scanning plane and the room corner.
3. The proposed calibration scheme is accurate. Camera calibration was based on the accurate coordinates of control points, which ensured the accuracy of the extrinsic parameters of the camera. Furthermore, robust linear fitting of LRF points was employed to locate the exact intersections between the LRF scanning plane and the room edges, which reduced the impact of noise of raw LRF points during LRF calibration.

Experiments on simulated and real data were conducted, which showed that the proposed scheme was accurate, precise, and robust. Comparison experiments demonstrated that the proposed scheme outperformed the state-of-the-art methods.

## 2. Methodology

### 2.1. Mathematic Framework

**Figure 2:**
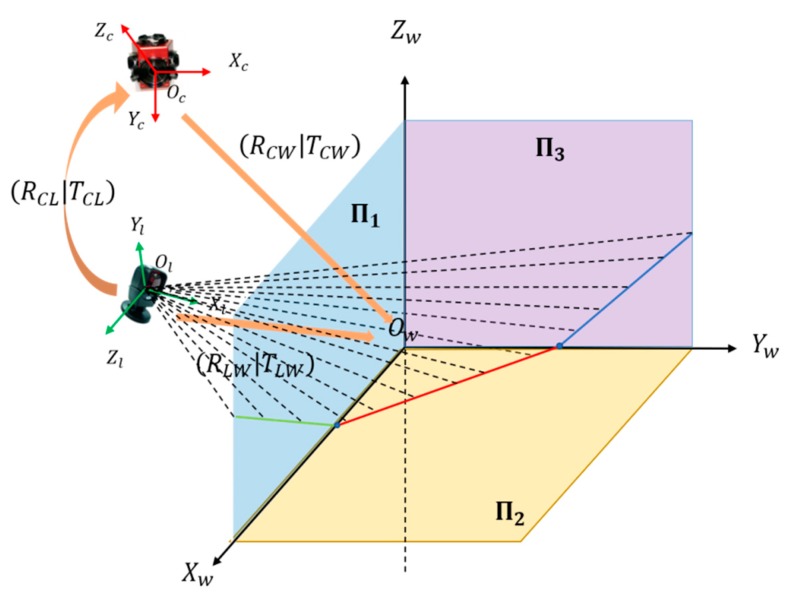
Configuration of the coordinate systems.

As illustrated in Figure 2, three coordinate systems of the camera, LRF, and control field were used. The control field coordinate system, denoted as (*O_w_–X_w_Y_w_Z_w_*), took the vertex of a room corner as the origin and three edges of the corner as the *x*-axis, *y*-axis, and *z*-axis, respectively. The camera coordinate system was denoted as (*O_c_–X_c_Y_c_Z_c_*). In our configuration, the camera’s optical center was the origin of the camera coordinate system, and the *x-o-y* plane was parallel to the imaging plane. We ignored the lens distortion for the rest of the paper and assumed that the images were already undistorted. The coordinate system of the 2D LRF was denoted as (*O_l_–X_l_Y_l_Z_l_*). We set the laser scanning center at its origin, and the scanning plane was denoted as the *x-o-z* plane.

Extrinsic calibration aims to obtain the extrinsic parameters that define the rigid relationship, that is, the rotation matrix and translation vector between two coordinate systems. Let (**R_CW_**|**T_CW_**) and (**R_LW_**|**T_LW_**) denote the extrinsic parameters of the camera and LRF coordinate systems, respectively, with respect to the control field coordinate system. For any point *P* in the scene, its coordinates in the control field, camera, and LRF coordinate systems are denoted as **P_w_** = (*X_w_, Y_w_, Z_w_*)*^T^*, **P_c_** = (*X_c_, Y_c_, Z_c_*)*^T^*, and **P_l_** = (*X_l_, Y_l_, Z_l_*)*^T^*, respectively. They satisfy the following relationships

(1) $$P_{c}=R_{CW}P_{w}+T_{CW},$$

(2) $$P_{l}=R_{LW}P_{w}+T_{LW}.$$

On the basis of Equations (1) and (2), the extrinsic parameters between the LRF and camera coordinate systems, denoted as (**R_CL_**|**T_CL_**), can be represented as

(3) $$\{\begin{array}{c} P_{c}=R_{CL}P_{l}+T_{CL}\\ R_{CL}=R_{CW}R_{Lw}^{-1}\\ T_{CL}=T_{CW}-R_{CL}T_{LW} \end{array}.$$

Equation (3) establishes the correspondence between the LRF and the camera through the common reference of the control field. Thus, the calibration of the extrinsic parameters between the camera and the LRF consists of two main steps, namely, LRF calibration with respect to the control field in Section 2.2 and camera calibration with respect to the control field in Section 2.3. 

### 2.2. Extrinsic Calibration of the LRF

To make the extrinsic calibration of LRF accurate and robust, the intersections of line features are employed to form a P3P problem, which can accurately locate the correspondences between the LRF and control field coordinate systems. A trirectangular trihedron formed by the room corner was used to perform a simplified P3P in the proposed scheme. 

**Figure 3:**
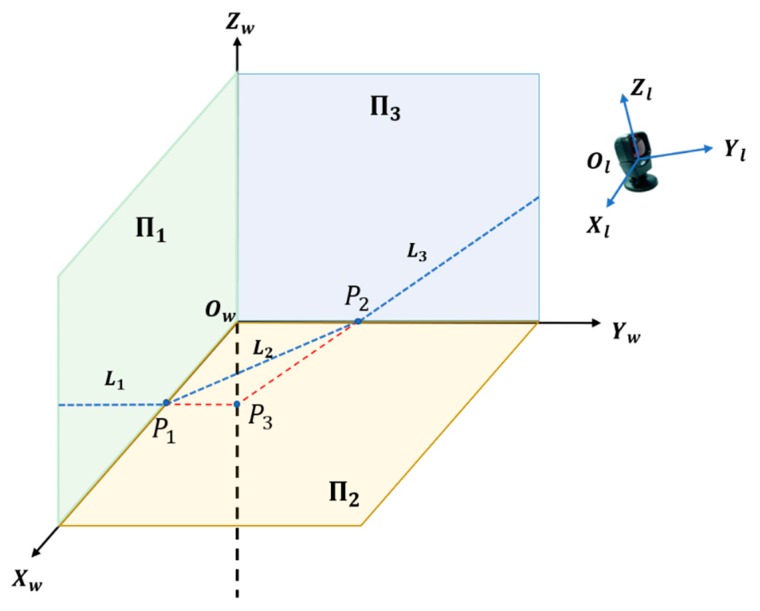
Schematic diagram of extrinsic calibration between the laser rangefinder (LRF) and control field.

As shown in Figure 3, the three planes of a room corner were denoted as Π_1_, Π_2_, and Π_3_, respectively. The scanning plane of the 2D LRF intersects the three planes at three line segments *L*_1_, *L*_2_, and *L*_3_ and intersects the three edges of the corner at *P*_1_, *P*_2_, and *P*_3_, respectively. 

In the LRF coordinate system, *L*_1_, *L*_2_, and *L*_3_ can be obtained by linearly fitting the laser range points of the segments. The intersection points *P*_1_, *P*_2_, and *P*_3_ are calculated based on the cross-product of the three line segments, that is

(4) $$\{\begin{array}{c} P_{1}=L_{1}\times L_{2}\\ P_{2}=L_{2}\times L_{3}\\ P_{3}=L_{1}\times L_{3} \end{array},$$

where × denotes the cross-product of two vectors. The three intersection points *P*_1_, *P*_2_, and *P*_3_ and the control field’s origin *O_w_* constitute a right triangle pyramid with the origin as the vertex. Side lengths *d*_1_, *d*_2_, and *d*_3_ of the triangle pyramid base can be calculated by

(5) $$\{\begin{array}{c} d_{1}=\left\|P_{1}-P_{2}\right\|\\ d_{2}=\left\|P_{1}-P_{3}\right\|\\ d_{3}=\left\|P_{2}-P_{3}\right\| \end{array},$$

where ║∙║ denotes the 2-norm distance between two points. Lengths *O_w_P*_1_, *O_w_P*_2_, and *O_w_P*_3_ are denoted as *λ*_1_, *λ*_2_, and *λ*_3_, respectively. Considering that the three edges of a room corner are perpendicular to each other, we have Equation (6) for the right triangle pyramid *O_w_*–*P*_1_*P*_2_*P*_3_.

(6) $$\{\begin{array}{c} \lambda_{1}^{2}+\lambda_{2}^{2}=d_{1}^{2}\\ \lambda_{1}^{2}+\lambda_{3}^{2}=d_{2}^{2}\\ \lambda_{2}^{2}+\lambda_{3}^{2}=d_{3}^{2} \end{array}.$$

There are eight feasible solutions for traditional P3P [9]. However, considering that Equation (6) is simplified without any angle and the length of the edges are greater than zero, we have the following unique solution

(7) $$\{\begin{array}{c} \lambda_{1}=\sqrt{\left(d_{1}^{2}+d_{2}^{2}-d_{3}^{2}\right)/2}\\ \lambda_{2}=\sqrt{\left(d_{1}^{2}+d_{3}^{2}-d_{2}^{2}\right)/2}\\ \lambda_{3}=\sqrt{\left(d_{2}^{2}+d_{3}^{2}-d_{1}^{2}\right)/2} \end{array}.$$

For the control field coordinate system, let *Q*_1_, *Q*_2_, and *Q*_3_ denote the three points that correspond to three intersection points *P*_1_, *P*_2_, and *P*_3_ in the LRF coordinate system. Considering that they are located on the three axes of the control field coordinate system and their distances away from origin *O_w_* are known by Equation (7), *Q*_1_, *Q*_2_, and *Q*_3_ can be expressed as

(8) $$\{\begin{array}{c} Q_{1}=(\lambda_{1},0,0)\\ Q_{2}=(0,\lambda_{2},0)\\ Q_{3}=(0,0,-\lambda_{3}) \end{array}.$$

**Figure 4:**
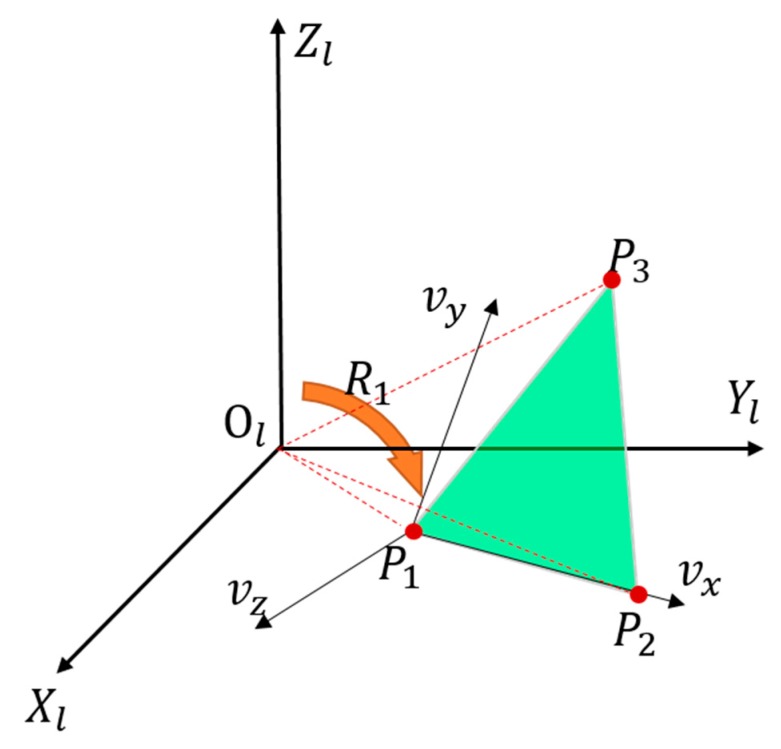
Schematic diagram of the three-point registration problem.

The coordinates of (*P*_1_, *P*_2_, *P*_3_) and (*Q*_1_, *Q*_2_, *Q*_3_) are the coordinates of the same points under two coordinate systems. Thus, solving the extrinsic parameters, i.e., the rotation matrix and translation vector, is actually a three-point registration problem of two coordinate systems [25]. As illustrated in Figure 4, let *P*_1_, *P*_2_, and *P*_3_ of the LRF coordinates form a spatial coordinate system with point *P*_1_ as the origin, and the three points lie on the *v_x_*-*P*_1_-*v_y_* plane. The axis *v_z_* is perpendicular to the *v_x_*-*P*_1_-*v_y_* plane. The rotation matrix of this coordinate system with respect to the LRF coordinate system is denoted as **R_1_**. In the same way, the same coordinate system of *v_x_*-*v_y_*-*v_z_* can be represented with the three equivalent points *Q*_1_, *Q*_2_, and *Q*_3_ of the control field coordinates, and rotation matrix **R_2_** of this coordinate system can be obtained with respect to the control field coordinate system. With the use of the *v_x_*-*v_y_*-*v_z_* as an intermediate coordinate system, we can calculate the rotation matrix between the LRF and the control field coordinate system.

(9) $$R_{LW}=R_{1}R_{2}^{-1}.$$

Then, the translation vector is easily acquired based on the correspondence between (*P*_1_, *P*_2_, *P*_3_) and (*Q*_1_, *Q*_2_, *Q*_3_).

(10) $$T_{LW}=\sum_{i=1}^{3}\left(P_{i}-R_{LW}Q_{i}\right)/3.$$

**Figure 5:**
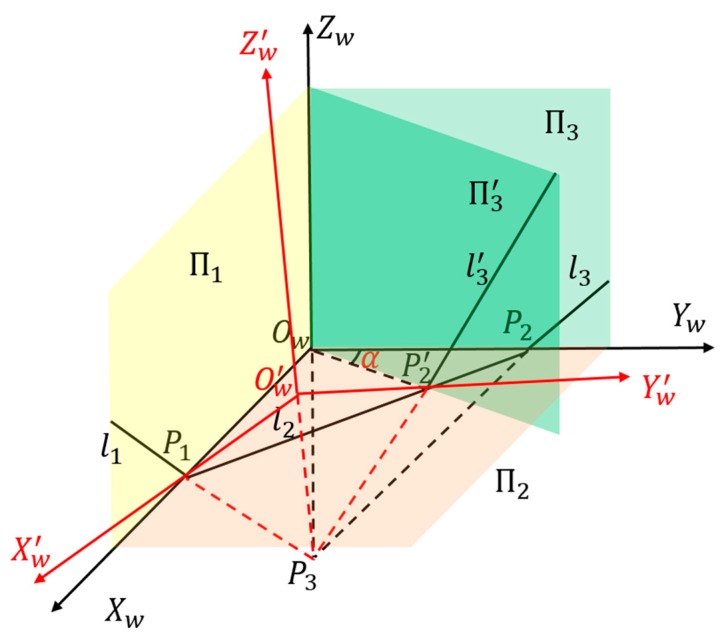
Schematic diagram of the impact of the deviation from orthogonality.

The trirectangular trihedron formed by the room corner is used in the extrinsic calibration of the LRF. The room corner may not be perfectly trirectangular, i.e., the angle formed by two planes has a deviation *α* from 90°. As shown in Figure 5, in an ideal situation, a trirectangular trihedron is formed by three planes П_1_, П_2_, and П_3_. The scanning plane of the 2D LRF intersects the three planes at the three line segments *l*_1_, *l*_2_, and *l*_3_, and intersects the three edges of the trirectangular trihedron at *P*_1_, *P*_2_, and *P*_3_, respectively. Rotating П_3_ around *Z_w_* to ${П}_{3}^{\prime }$, the trihedron formed by П_1_, П_2_, and ${П}_{3}^{\prime }$ is an imperfect trirectangular trihedron due to the rotation angle *α*. The scanning plane of the LRF intersects the three planes П_1_, П_2_, and ${П}_{3}^{\prime }$ with the three line segments *l*_1_, *l*_2_, and $l_{3}^{\prime }$, and intersects the three edges of the new trihedron at *P*_1_, $P_{2}^{\prime }$, and *P*_3_, respectively.

In an ideal situation, the right triangle pyramid *O_w_*–*P*_1_*P*_2_*P*_3_ is used to solve a simplified P3P and finally obtain the rotation and translation of the LRF coordinate system with respect to the *O_w_–X_w_Y_w_Z_w_*, which is the extrinsic parameters of LRF. Due to the deviation *α*, the ideal intersection point *P*_2_ moves to $P_{2}^{\prime }$, and the new triangular pyramid *O_w_*–*P*_1_$P_{2}^{\prime }$*P*_3_ is not a right triangle pyramid. However, we still solve a simplified P3P, which means that a right triangle pyramid $O_{w}^{\prime }$–*P*_1_$P_{2}^{\prime }$*P_3_* is formed, and thus a new coordinate system $O_{w}^{\prime }–X_{w}^{\prime }Y_{w}^{\prime }Z_{w}^{\prime }$ is built. In this situation, the calculated extrinsic parameters of LRF is actually the rotation and translation of LRF with respect to $O_{w}^{\prime }–X_{w}^{\prime }Y_{w}^{\prime }Z_{w}^{\prime }$. Therefore, rotation and translation between *O_w_–X_w_Y_w_Z_w_* and $O_{w}^{\prime }–X_{w}^{\prime }Y_{w}^{\prime }Z_{w}^{\prime }$ equals the extrinsic calibration error of LRF with respect to control field caused by the deviation *α* from 90°. The larger the deviation *α* is, the larger the difference between *P*_1_*P*_2_ and *P*_1_$P_{2}^{\prime }$ is, the larger the calibration error.

It should be noted that one scan of the room corner is sufficient to determine the extrinsic parameters between the LRF and the control field. The trirectangular trihedron formed by the room corner makes the P3P solution unique, as derived in Equation (7). In addition, degeneration problems in P3P caused by two parallel planes and a danger cylinder [11] are avoided by using the room corner. The intersections of three lines accurately locate the correspondences between the range data and the control field, which ensures the accuracy of LRF calibration. Besides, calibration does not directly use the raw LRF range data as in Zhang’s method [5]. Instead, calibration uses linear features that are robustly derived from the raw LRF range data, which reduces the impact of noises on the extrinsic calibration of the LRF.

### 2.3. Extrinsic Calibration of the Camera

Extrinsic calibration of the camera determines its extrinsic parameters with respect to the control field. The extrinsic and intrinsic parameters determine the position and imaging plane of the camera in the control field. The intrinsic parameters of the camera are denoted as (*x*_0_, *y*_0_, *f*), where (*x*_0_, *y*_0_) denotes the principal point, and *f* is the focal length. The extrinsic parameters of the camera can be denoted as (**R_CW_**, **T_CW_**), where

(11) $$R_{CW}=R_{\varphi }R_{\omega }R_{\kappa }=\left[\begin{array}{ccc} a_{1} & b_{1} & c_{1}\\ a_{2} & b_{2} & c_{2}\\ a_{3} & b_{3} & c_{3} \end{array}\right],$$

(12) $$T_{CW}={\left[X_{S},Y_{S},Z_{S}\right]}^{T},$$

where **R_CW_** represents a 3 × 3 rotation matrix formed by rotation angle (*φ*, *ω*, *κ*) between the three axes of the camera and control field coordinate systems, and **T_CW_** is a 3 × 1 translation vector that represents the origin of the camera coordinate system in the control field coordinate system. The control field coordinate system is shown in Section 2.1. According to imaging principle, camera origin **S** (*X_S_, Y_S_, Z_S_*)*^T^* and point **P** (*X, Y, Z*)*^T^* in the control field and the corresponding image point **p**(*x*, *y*) of **P** are collinear. The collinearity can be represented as

(13) $$\left\{ \begin{array}{l} x-x_{0}=-f\frac{a_{1}(X-X_{S})+b_{1}(Y-Y_{S})+c_{1}(Z-Z_{S})}{a_{3}(X-X_{S})+b_{3}(Y-Y_{S})+c_{3}(Z-Z_{S})}\\ y-y_{0}=-f\frac{a_{2}(X-X_{S})+b_{2}(Y-Y_{S})+c_{2}(Z-Z_{S})}{a_{3}(X-X_{S})+b_{3}(Y-Y_{S})+c_{3}(Z-Z_{S})} \end{array}\right..$$

Since the camera used for calibration in our integrated sensor is a fisheye camera with relatively large distortion, we first rectify the distortion before the extrinsic calibration in order to achieve a better calibration result. Thus, Equation (13) only contains intrinsic and extrinsic parameters. Introducing independent parameters *l_i_* (*i* = 1, 2 … 11), Equation (13) can be re-formulated as follows

(14) $$\left\{ \begin{array}{l} x+\frac{l_{1}X+l_{2}Y+l_{3}Z+l_{4}}{l_{9}X+l_{10}Y+l_{11}Z+1}=0\\ y+\frac{l_{5}X+l_{6}Y+l_{7}Z+l_{8}}{l_{9}X+l_{10}Y+l_{11}Z+1}=0 \end{array}\right.,$$

where *l_i_* (*i* = 1, 2 … 11) are the functions of the intrinsic and extrinsic parameters of the camera, as shown in Equation (15).

(15) $$\left[\begin{array}{cccc} l_{1} & l_{2} & l_{3} & l_{4}\\ l_{5} & l_{6} & l_{7} & l_{8}\\ l_{9} & l_{10} & l_{11} & 1 \end{array}\right]=\left[\begin{array}{cccc} f & 0 & x_{0} & 0\\ 0 & f & y_{0} & 0\\ 0 & 0 & 1 & 0 \end{array}\right]\left[\begin{array}{cc} R_{CW} & T_{CW}\\ 0^{T} & 1 \end{array}\right].$$

These linear constraints can be solved through DLT [23,24]. To avoid non-linear computation, below we use the classical DLT in the proposed scheme. The revised DLT with two constraints [26] can be used for more rigorous extraction of intrinsic and extrinsic parameters if higher accuracy is required. 

Equation (14) is rearranged by eliminating its denominator, as shown below

(16) $$\{\begin{array}{c} l_{1}X+l_{2}Y+l_{3}Z+l_{4}+0+0+0+0+xl_{9}X+xl_{10}Y+xl_{11}Z+x=0\\ 0+0+0+0+l_{5}X+l_{6}Y+l_{7}Z+l_{8}+yl_{9}X+yl_{10}Y+yl_{11}Z+y=0 \end{array}.$$

The initial value of 11 linear transformation parameters *l_i_* (*i* = 1, 2 … 11) can be calculated with Equation (16) by using six control points. To obtain more accurate and robust results, an iterative calculation was performed. Denote the correction of the observation of image point **p**(*x*, *y*) as (*v_x_*, *v_y_*), and let

(17) $$A=l_{9}X+l_{10}Y+l_{11}Z+1.$$

Then Equation (14) can be written as

(18) V=ML−W,

where

$$\begin{array}{l} V=\left[\begin{array}{c} v_{x}\\ v_{y} \end{array}\right]\\ M=\left[\begin{array}{ccccccccccc} \frac{X}{A} & \frac{Y}{A} & \frac{Z}{A} & \frac{1}{A} & 0 & 0 & 0 & 0 & \frac{xX}{A} & \frac{xY}{A} & \frac{xZ}{A}\\ 0 & 0 & 0 & 0 & \frac{X}{A} & \frac{Y}{A} & \frac{Z}{A} & \frac{1}{A} & \frac{yX}{A} & \frac{yY}{A} & \frac{yZ}{A} \end{array}\right]\\ L={\left[\begin{array}{ccccccccccc} l_{1} & l_{2} & l_{3} & l_{4} & l_{5} & l_{6} & l_{7} & l_{8} & l_{9} & l_{10} & l_{11} \end{array}\right]}^{T}\\ W=\left[\begin{array}{c} \frac{x}{A}\\ \frac{y}{A} \end{array}\right] \end{array}$$

Least squares [27] was used to calculate a new value of *l_i_* (*i* = 1, 2 … 11) with a large number of control points in the control field.

(19) $$L=(M^{T}M{)}^{-1}M^{T}W.$$

The translation vector can be solved after coefficients *l_i_* (*i* = 1, 2 … 11) are obtained. Considering that **R_CW_** is a unit orthogonal matrix, we deduced that the translation vector of the extrinsic parameters satisfies the following relationship from Equation (15)

(20) $$\left\{ \begin{array}{l} l_{1}X_{S}+l_{2}Y_{S}+l_{3}Z_{S}=-l_{4}\\ l_{5}X_{S}+l_{6}Y_{S}+l_{7}Z_{S}=-l_{8}\\ l_{9}X_{S}+l_{10}Y_{S}+l_{11}Z_{S}=-1 \end{array}\right.,$$

where the three elements of the translation vector form three independent linear equations.

Similarly, we can derive the relationship between coefficients *l_i_* (*i* = 1, 2 … 11) and nine parameters of the rotation matrix from Equation (15). The three angles that compose the rotation matrix can be obtained as follows

(21) $$\left\{ \begin{array}{l} \tan\phi =-\frac{a_{3}}{c_{3}}=-\frac{l_{9}}{l_{11}}\\ \sin\omega =-b_{3}=-\frac{l_{10}}{\sqrt{l_{9}{}^{2}+l_{10}{}^{2}+l_{11}{}^{2}}}\\ \tan\kappa =\frac{b_{1}}{b_{2}}=\frac{x_{0}l_{10}+l_{2}}{y_{0}l_{10}+l_{6}} \end{array}\right..$$

Start with initial values, values of A, *l_i_* (*i* = 1, 2 … 11), (*X_S_, Y_S_, Z_S_*), and (*φ*, *ω*, *κ*) were updated based on Equations (17), (19), (20), and (21), respectively. The iterative procedure ends when it satisfies that the differences of *l_i_* (*i* = 1, 2 … 11) and the differences of *X_S_, Y_S_,* and *Z_S_* calculated in two successive iterations are all less than the corresponding thresholds. Finally, (**R_CW_**|**T_CW_**) between the camera and the control field coordinate systems are obtained through the (*X_S_, Y_S_, Z_S_*) and (*φ*, *ω*, *κ*) calculated in the last iteration.

From the above analysis, we can draw the following characteristics about the control field based camera calibration. First, the evenly distributed control points in the control field avoid the degeneration problems in camera calibration caused by coplane or collinearity of the control points [28]. Second, the high precision of the coordinates of control points in the control field and in the image ensures the collinear condition for DLT calculation, which results in accurate camera calibration. Furthermore, a large number of control points provide redundant observations, which makes the extrinsic calibration of the camera robust and accurate. Besides, it requires only one shot at the control field to obtain the extrinsic parameters of the camera with respect to the control field.

Finally, the extrinsic parameters between the camera and the LRF can be obtained using Equation (3) once the extrinsic parameters of the LRF (Section 2.2) and the extrinsic parameters of the camera (Section 2.3) are respectively determined with respect to the control field. As demonstrated in Section 2.2 and Section 2.3, the proposed scheme provides accurate and robust results for the extrinsic calibration of the LRF and the extrinsic calibration of the camera, respectively. Thus, the extrinsic calibration between the camera and the LRF is accurate and robust. In addition, the entire calibration requires only one shot at the control field to simultaneously collect the data for extrinsic calibration of the LRF and the camera.

## 3. Experiments

We conducted experiments on simulated and real data to verify the accuracy, precision and robustness of the proposed calibration scheme. First, the simulated data with ground truth were used to evaluate the performance of the proposed scheme under different noise levels. Second, a sensor system with a 2D LRF and a camera was calibrated and evaluated in indoor and outdoor scenes to validate the effectiveness of the proposed scheme in practical applications.

### 3.1. Experiments with Simulated Data

For the entire process of extrinsic calibration, the factors that affect accuracy are the number of laser points in each plane, the angle between the three planes in the trihedron, the existing noises in the laser points, the number of control points, the distribution of control points, and the existing noises in the control points. Hu et al. [17] performed statistical experiments on the impact of the number of laser points in each plane and the angle between the three planes. Chen et al. [28] conducted statistical experiments on the impact of the number and the distribution of the control points. Thus, we set these four factors as suggested. Considering that the coordinates of the control points in the control field are precisely measured, we test the performance of the proposed scheme in terms of different image noise levels, different laser range noise levels, and outliers in the image and laser range.

In the simulated experiments, the camera and the LRF were simulated based on the parameters of real sensors. The focal length of the camera was set to 12 mm, and the resolution was 4608 × 3456 pixels. The principal point of the camera was located at the center of the image. The camera was set without lens distortion. For the 2D LRF, the laser scanning scope was 270°, and the angular resolution was 0.25°. The photogrammetric control field was modeled as a 3D cuboid. The three perpendicular edges of the cuboid and the vertex of the three edges formed the coordinate system (*O_w_–X_w_Y_w_Z_w_*). A total of 360 control points was evenly distributed in the 2.5 × 2.5 × 2.7 m^3^ cuboid. The spacing of control points in the *x*-axis, *y*-axis, and *z*-axis directions were 50, 50, and 30 mm, respectively. The image data were generated using an ideal pinhole imaging model. The LRF points of range data were generated by shooting laser rays on the photogrammetric control field. Gaussian noises with zero mean and different noise levels were added to offset the pixel coordinates of the image, which are due to the ambiguity and low contrast around feature pixels. Similarly, the range data of LRF points were added with zero-mean Gaussian noise at different levels to simulate their uncertainty in distance measurements.

In the simulated experiments, the extrinsic parameters of rotation (in Euler angles) and translation between the LRF and the camera were set as follows

(22) $$\{\begin{array}{c} \psi_{t}=[15^{\circ },2^{\circ },0.1^{\circ }]\\ T_{t}={\left[10mm,600mm,20mm\right]}^{T} \end{array},$$

where row vector **ψ_t_** represents the three Euler angles of rotation, and column vector **T_t_** represents the translation. These parameters are used as ground truth for the simulated experiments to verify the accuracy, precision, and robustness of the proposed scheme under different noises. 

The errors of rotation and translation are measured to quantitatively evaluate the performance of the results, which are expressed as follows

(23) $$\{\begin{array}{c} E_{r_{i}}=\cos^{-1}\frac{r_{i_{t}}r_{i}^{T}}{∥r_{i_{t}}∥\times ∥r_{i}∥}\\ E_{T}=∥T-T_{t}∥ \end{array},$$

where **r_i_** (*i* = 1, 2, 3) are the three column vectors of the rotation matrix calculated by the proposed scheme, and $r_{i_{t}}$ (*i* = 1, 2, 3) are the three column vectors of the rotation matrix calculated by **ψ_t_**. $E_{r_{i}}$ (*i* = 1, 2, 3) measures the deviation angle between the two column vectors, which are used to quantitatively evaluate the performance of rotation. The smaller $E_{r_{i}}$ is, the more accurate the calibration of rotation. **T** is the translation vector calculated by the proposed scheme. *E_T_* denotes the 2-norm of the difference of the calculated translation vectors and the ground truth, which is used to quantitatively evaluate the performance of translation. Similarly, the smaller *E_T_* is, the more accurate the calibration of translation.

#### 3.1.1. Performance in Terms of Image Noise

**Figure 6:**
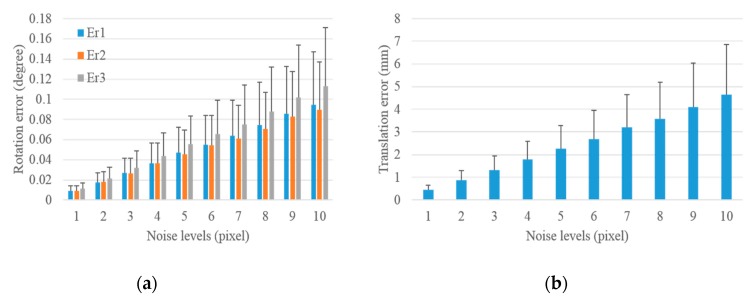
Rotation and translation errors for the extrinsic calibration of camera with respect to the control field under increasing image noise levels. (**a**) Rotation error and (**b**) translation error.

**Figure 7:**
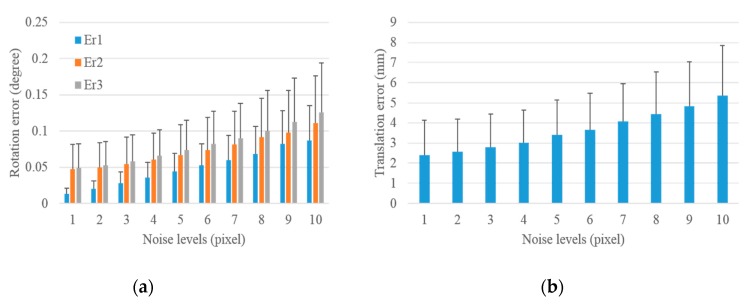
Rotation and translation errors for the extrinsic calibration of LRF with respect to the camera under increasing image noise levels. (**a**) Rotation error and (**b**) translation error.

We added different levels of noise to the image and calculated the mean and standard deviation of translation and rotation errors under each noise level to verify the accuracy and precision of the proposed scheme with respect to image noise. A total of 1000 groups of experiments were independently performed under each noise level. For the LRF, we added 3 mm Gaussian noise with zero-mean to the laser range. Noise levels from 1 pixel to 10 pixels were added to the image data to test the performance of the scheme with respect to image noise. The translation and rotation errors under different noise levels for the extrinsic camera calibration with respect to the control field and the LRF are demonstrated in Figure 6 and Figure 7.

The rotation and translation errors for the extrinsic calibration of the camera with respect to the control field are shown in Figure 6. The bar in the histogram represents the mean of errors of 1000 independent experiments, and the error bar above the bar indicates the standard deviation of errors. The rotation error under each noise level was determined based on three column vector errors $E_{r_{1}}$, $E_{r_{2}}$, and $E_{r_{3}}$, which is less than the maximum of the three errors. As shown in Figure 6, the mean of rotation and translation errors increase with the increase in image noise levels from 1 pixel to 10 pixels. The mean of the rotation errors increased from 0.009°, 0.009°, and 0.011° to 0.095°, 0.090°, and 0.113°, and the mean of the translation errors increased from 0.449 mm to 4.652 mm. This indicates that the camera calibration was accurate even under large image noise level of 10 pixels. In addition, the standard deviations of rotation and translation errors increased with the increase in noise levels from 1 pixel to 10 pixels, which indicates that the results of the camera calibration worsen with the increase in noise levels. However, Figure 6 shows that the standard deviations of rotation errors increased from 0.005°, 0.005°, and 0.006° to 0.052°, 0.047°, and 0.058°, and that of translation errors increased from 0.208 mm to 2.203 mm, thereby indicating that the camera calibration is precise.

After analyzing the impact of image noises on the extrinsic calibration of camera with respect to the control field in Figure 6, the entire process of the proposed scheme was implemented to analyze the effect of image noises on the extrinsic calibration of the camera with respect to LRF. The statistical results are shown in Figure 7. The mean and standard deviations of rotation and translation errors increased with the increase in image noise levels from 1 pixel to 10 pixels. The mean of rotation errors increased from 0.013°, 0.047°, 0.049° to 0.087°, 0.111°, and 0.126°, and the standard deviations increased from 0.007°, 0.034°, 0.034° to 0.048°, 0.065°, and 0.068°. The mean of translation errors increased from 2.412 mm to 5.367 mm, and the standard deviations increased from 1.715 mm to 2.489 mm with the increase in noise levels. The above analysis shows that the mean and standard deviations of translation and rotation errors were small even under the noise of 10 pixels, which demonstrates that the proposed scheme was accurate and precise for the extrinsic camera calibration with respect to LRF under the effect of image noises. In addition, the mean and standard deviations of these errors increased slightly with the increase in image noises from 1 pixel to 10 pixels, thereby verifying that the number of control points provides redundant observations and makes the proposed scheme insensitive to image noises. The rotation and translation errors changed remarkably in Figure 7 compared with Figure 6, thereby indicating that the addition of laser range noise obviously impacts the extrinsic camera calibration with respect to LRF.

#### 3.1.2. Performance in Terms of Laser Range Noise

**Figure 8:**
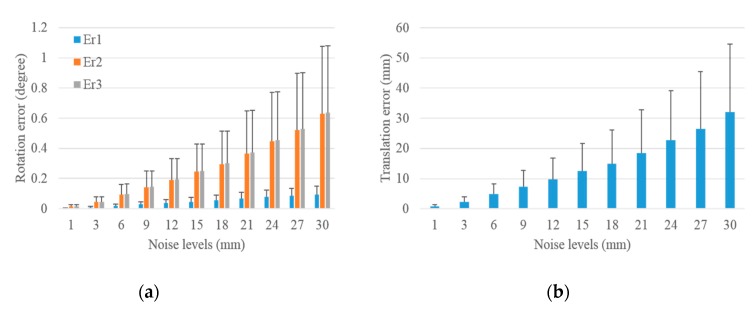
Rotation and translation errors for the extrinsic calibration of LRF with respect to the control field under increasing range noise levels. (**a**) Rotation error and (**b**) translation error.

**Figure 9:**
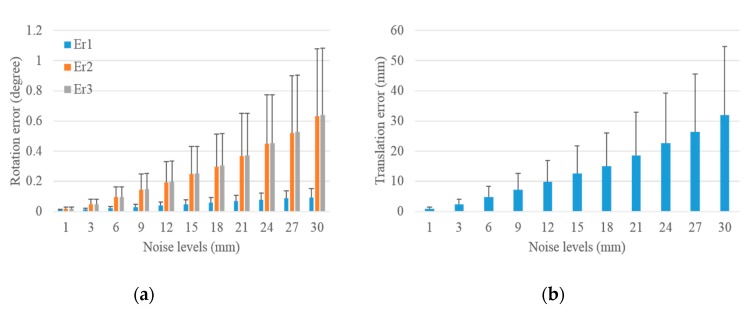
Rotation and translation errors for the extrinsic calibration of LRF with respect to the camera under increasing range noise levels. (**a**) Rotation error and (**b**) translation error.

Similar simulated experiments were performed to evaluate the accuracy and precision of the proposed scheme with respect to laser range noise. One-pixel Gaussian noise was added in the image, and noise levels from 1 mm to 30 mm were added to the laser range to test the performance of the proposed scheme with respect to laser range noise. A total of 1000 groups of experiments were independently performed under each level of range noise. The statistical results under different noise levels for the extrinsic LRF calibration with respect to the control field and the camera are shown in Figure 8 and Figure 9, respectively.

As shown in Figure 8, the mean and standard deviations of rotation and translation errors for the extrinsic calibration of LRF with respect to the control field increase with the increase in the laser range noise levels. Under 1 mm noise, the mean and standard deviation of rotation errors in the three columns were 0.003°, 0.014°, 0.014° and 0.002°, 0.011°, 0.011°, respectively, and under 30 mm noise, they were 0.092°, 0.632°, 0.639° and 0.057°, 0.445°, 0.441°, respectively. The mean of translation errors increased from 0.714 mm to 32.045 mm, and the standard deviation of translation errors increased from 0.555 mm to 22.653 mm. This finding demonstrates that the extrinsic LRF calibration with respect to the control field was accurate and precise to range noise.

The proposed scheme was performed with the range noise from 1 mm to 30 mm to evaluate the impact of range noise on the extrinsic LRF calibration with respect to the camera. The statistical results are shown in Figure 9. The mean and standard deviation of rotation and translation errors increased with the increase in the range noise levels. The minimum mean and standard deviation of rotation errors in the three columns were 0.009°, 0.017°, 0.019° and 0.005°, 0.011°, 0.011°, respectively, and the corresponding maximum values were 0.093°, 0.632°, 0.639° and 0.057°, 0.446°, 0.441°, respectively. The mean of translation errors increased from 0.870 mm to 32.055 mm, and the standard deviation of translation errors increased from 0.521 mm to 22.658 mm. This finding indicates that the extrinsic LRF calibration with respect to the control field was accurate and precise with respect to range noise. In addition, the rotation and translation errors were approximately the same not only in the increasing trend, as shown in Figure 8 and Figure 9, but also in the above given values. This condition demonstrates that the addition of image noise had a slight impact on the extrinsic calibration of LRF with respect to camera.

The above analysis suggests that the addition of range noise has more influence than the addition of image noise on the extrinsic calibration between camera and LRF. In addition, the rotation and translation errors increased more rapidly with the increase in the laser range noise than with the increase in image noise, as shown in Figure 7 and Figure 9. Thus, we infer that the laser range noise had more effect than image noise on the extrinsic calibration between the camera and the LRF. 

#### 3.1.3. Performance in Terms of Outliers

**Figure 10:**
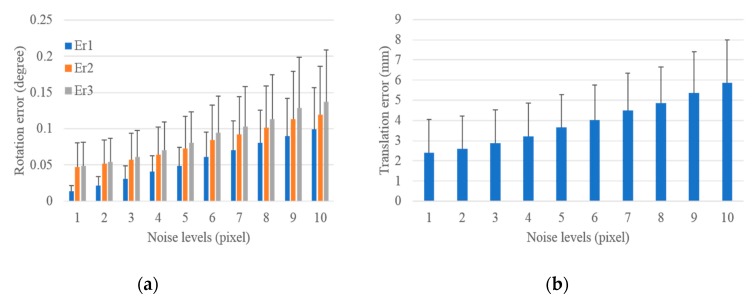
Rotation and translation errors for the extrinsic calibration of LRF with respect to camera with outliers added in image. (**a**) Rotation error and (**b**) translation error.

**Figure 11:**
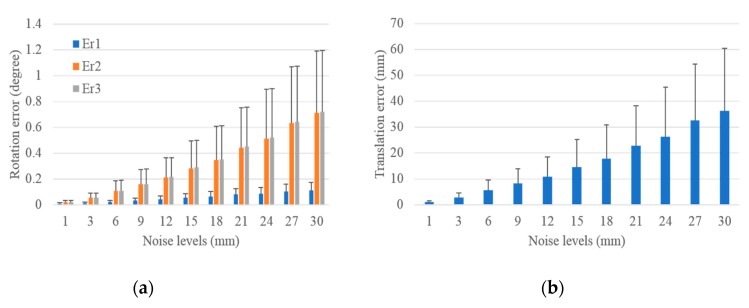
Rotation and translation errors for the extrinsic calibration of LRF with respect to the camera with outliers added in the laser range. (**a**) Rotation error and (**b**) translation error.

Outliers were added to the image and laser range, respectively, to test the robustness of the proposed scheme. The noise levels were set the same as in Figure 7 and Figure 9. Under each noise level, 3% outliers were added, and outliers were set to a Gaussian noise with a mean equaling triple the noise level, and a standard deviation equaling the noise level. A total of 1000 groups of experiments were independently performed under each noise level, and the mean and standard deviation of translation and rotation errors were calculated. The translation and rotation errors for the extrinsic LRF calibration with respect to the camera with outliers in image noise and laser range noise are demonstrated in Figure 10 and Figure 11.

As shown in Figure 10, although 3% of outliers were added in the image, the rotation and translation errors remained small. Compared with Figure 7, in which no outliers were added, they had the same increased trend. Under the noise level of 1 pixel, the mean and standard deviation of the rotation and translation errors were almost the same with that in Figure 7. Under the noise level of 10 pixels, compared with the results shown in Figure 7, it had a 0.013°, 0.009°, and 0.011° increase in the mean of rotation errors, 0.009°, 0.002°, and 0.004° increase in the standard deviation of rotation errors, 0.485 mm and 0.220 mm increase in the mean and the standard deviation of translation errors, respectively. The absolute value and increase of the rotation and translation errors demonstrate that the proposed scheme was robust with respect to the outliers in the image, and the outliers had almost no impact on the calibration results under low noise levels.

The performance of the proposed scheme with respect to the outliers in the laser range is shown in Figure 11. Compared with the errors in Figure 9, in which no outliers were added, it had the same increasing trend and a small increase in the values. Besides, it had a 0°, 0.002°, and 0.002° increase in the mean of rotation errors, 0°, 0.002°, and 0.002° increase in the standard deviation of rotation errors, 0.125 mm and 0.059 mm increase in the mean and the standard deviation of translation errors under the noise level of 1 mm, and a 0.007°, 0.078°, and 0.080° increase in the mean of rotation errors, 0.008°, 0.048°, and 0.047° increase in the standard deviation of rotation errors, 4.304 mm and 2.194 mm increase in the mean and the standard deviation of translation errors under the noise level of 30 mm. The above analysis reveals that the proposed scheme was robust with respect to the outliers in the laser range, and the robustness of the proposed scheme decreased with the increasing noise levels.

#### 3.1.4. Comparison Experiments

The proposed scheme was compared with the state-of-the-art methods proposed by Hu [17] and Zhang [5]. A total of 1000 groups of experiments were independently performed under each noise level. The mean of rotation and translation errors for the camera calibration with respect to LRF under different noise levels is shown in Table 1, and the standard deviations of rotation and translation errors are shown in Table 2. As shown in Table 1 and Table 2, the mean and standard deviations of rotation and translation errors of our scheme was better than that of Hu’s and Zhang’s methods under the same noise level. Moreover, as shown in Table 1, the maximum mean errors of our scheme were only greater than the minimum mean errors of Zhang’s method, and greater than the two minimum mean errors of Hu’s method. Besides, comparing the standard deviations of errors of the three methods in Table 2, we can conclude the same as for the mean errors. Therefore, the proposed scheme was more accurate and precise than Hu’s and Zhang’s methods.

### 3.2. Experiments with Real Data

**Figure 12:**
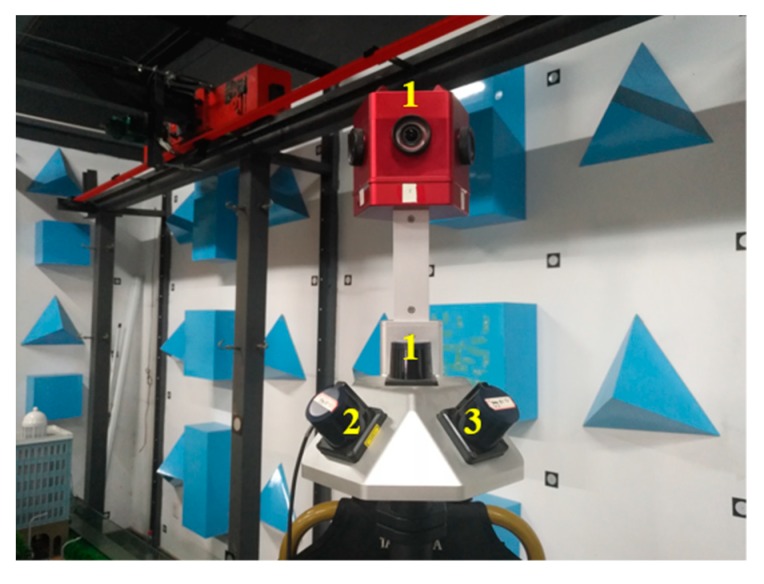
Integrated sensor composed of cameras and LRFs.

An integrated sensor composed of cameras and LRFs, as shown in Figure 12, was used to calibrate the extrinsic parameters by using the proposed scheme to verify its effectiveness in practical applications. Indoor and outdoor scenes were collected to evaluate the performance of the proposed scheme.

The integrated sensor in Figure 12 was composed of six cameras (placed on top of the system) and three LRFs (placed at the bottom of the system). As an example, the extrinsic parameters between camera 1 and LRF 1 in Figure 12 were calibrated in this experiment. The camera is Xiaoyi motion camera. It has an image resolution of 4608 × 3456 pixels, a focal length of 3.2 mm, and a field of view of 145°. The LRF is Hokuyo UTM-30LX-EM. It had a field of view of 270°, an angular resolution of 0.25°, and a measuring distance from 0.1 m to 30 m. The ranging accuracy was ± 30 mm for measuring distance from 0.1 m to 10 m, and ±50 mm for measuring distance from 10 m to 30 m. The coordinate system of the control field was built, as shown in Figure 1. The control field occupied a space of 3 × 5 m^2^. A large number of control points were evenly distributed at different heights of poles. As shown in Figure 1, three rows of poles and seven poles per row were found in the control field. Many control points were distributed on the walls around the room. The 3D coordinates of the control points in the control field coordinate system were previously measured by Sokkia NET 1005 total station, and the root mean square error in the coordinates was 0.1 mm. The three angles of the room corners in the photogrammetric control field were calculated by fitting three planes using the coordinates of the control points in three walls. The three angles were 90.176°, 90.220°, and 90.135°, respectively.

**Figure 13:**
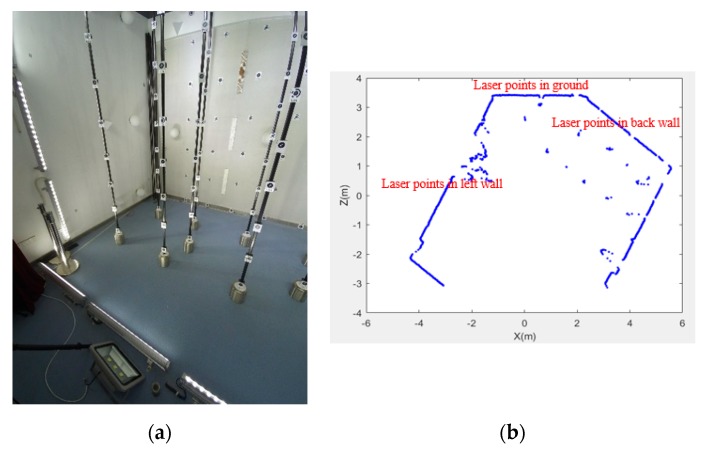
Image and range data for the extrinsic calibration between a camera and an LRF. (**a**) Image and (**b**) LRF range data.

To calibrate the extrinsic parameters between the camera 1 and LRF 1 in Figure 12, the image and range data of the two sensors were collected in the control field. Since the camera used was a fisheye camera, the distortion of the image was rectified in advance, and the image after the rectification is shown in Figure 13a. The image data captured a large number of control points, the ground plane, the left and back wall planes, and the left-bottom corner of the control field. As shown in Figure 13b, the LRF range data contained five lines, and the three lines marked by red text were the intersections of the laser scanning plane and the three neighboring planes of the left-bottom corner.

**Figure 14:**
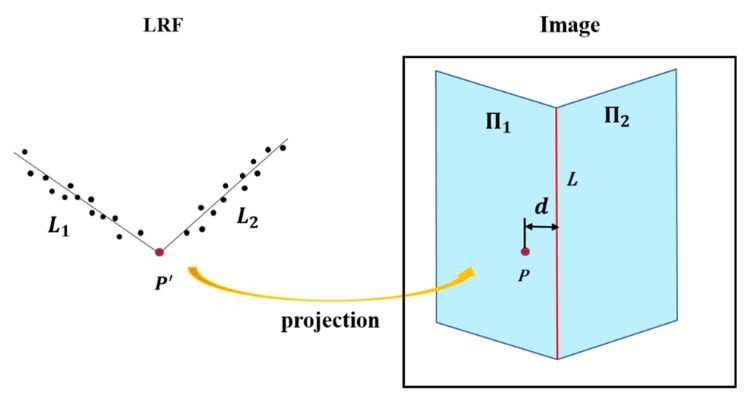
Schematic diagram of point-to-edge distance.

The extrinsic parameters between the camera and the LRF were calibrated using the proposed scheme, and the calibration results were evaluated qualitatively and quantitatively. Considering that the rigid transformation between two coordinate systems and the pin-hole geometric imaging transformation were linear, the lines formed by the intersections of laser scanning plane and planes in the scene remain as lines in the corresponding plane in the projected image. This feature was used for qualitative evaluation of the results. To quantitatively evaluate the result, point-to-edge distance was designed as demonstrated in Figure 14. 

As shown in Figure 14, the imaging of two intersecting planes are Π_1_ and Π_2_ in the image, and the intersection line of Π_1_ and Π_2_ is *L*. The lines fitted by the laser points on the two planes are *L*_1_ and *L*_2_, respectively. The intersection of *L*_1_ and *L*_2_ is *P*′, and *P* is the projection of *P*′ on the image. Point-to-edge distance is the distance from *P* to *L*. The smaller the point-to-edge distance is, the better the result. The image coordinates of *P* are denoted as (*x_p_, y_p_*), and the equation of *L* is *ax + by – c* = 0, we have the point-to-edge distance as follows

(24) $$d=\frac{\left\|ax_{p}+by_{p}-c\right\|}{\sqrt{a^{2}+b^{2}}}.$$

**Figure 15:**
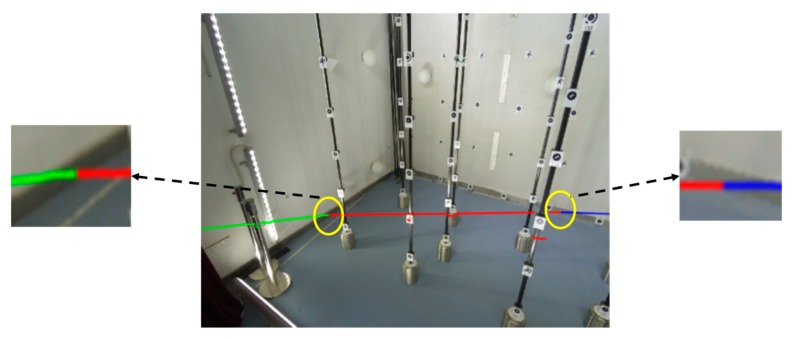
Projection of LRF range data on the image using the extrinsic parameters calibrated by our scheme.

Using the extrinsic parameters calibrated by the proposed scheme, Figure 15 shows the projection results of the laser points on the image using the data in Figure 13, where the green, red, and blue lines are composed of the projected LRF laser points of the left wall, ground, and back wall, respectively. The three lines were correctly located on the corresponding planes, and the two intersections were located on the intersection lines of the three planes. The point-to-edge distances in the image were calculated to evaluate the calibration accuracy. The left and right point-to-edge distances were 0.5 and 0.3 pixels, respectively, thereby showing that the extrinsic calibration using the proposed scheme was sufficiently accurate.

**Figure 16:**
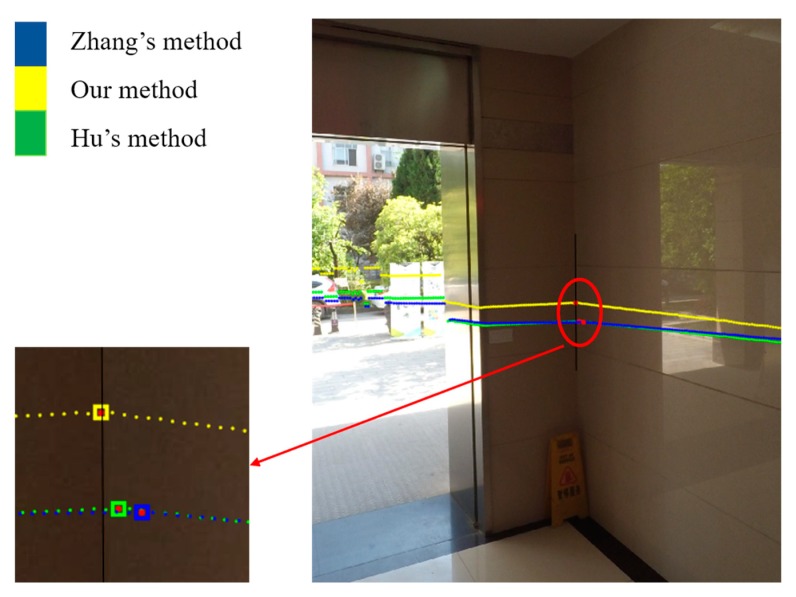
Projection of LRF range data on the image (indoor).

Typical indoor and outdoor scenes were adopted to verify the effectiveness of the proposed scheme and the accuracy of extrinsic calibration results. The calibration using Zhang’s method [5] and Hu’s method [17] were performed for comparison. The indoor scene is shown in Figure 16. We used two walls to help verify the accuracy of the calculated extrinsic parameters. The integrated sensor was about 3 m away from the walls and shot the walls almost vertically. The camera and the LRF captured the walls at the same time. The LRF laser points were projected on the image using our, Zhang’s, and Hu’s extrinsic parameters, which were represented by yellow, blue, and green points, respectively, in Figure 16. The yellow line was almost parallel to the adjacent horizontal line in the wall, while the green and blue lines almost intersected the adjacent horizontal line. This condition demonstrates that the rotation parameters calculated by our calibration scheme was more accurate because the sensor shot the walls almost vertically. Then, we extracted the linking edge of the two walls in the image, as shown by the black line in Figure 16. The intersection points, as shown by the red points in Figure 16, were obtained by projecting the intersection points of two fitted laser lines in the LRF coordinate system on the image. Finally, we calculate the point-to-edge distance. The point-to-edge distance using our, Zhang’s, and Hu’s extrinsic parameters were 0.79, 27.60, and 11.13 pixels, respectively. This finding indicates that our calibration scheme was much more accurate. 

**Figure 17:**
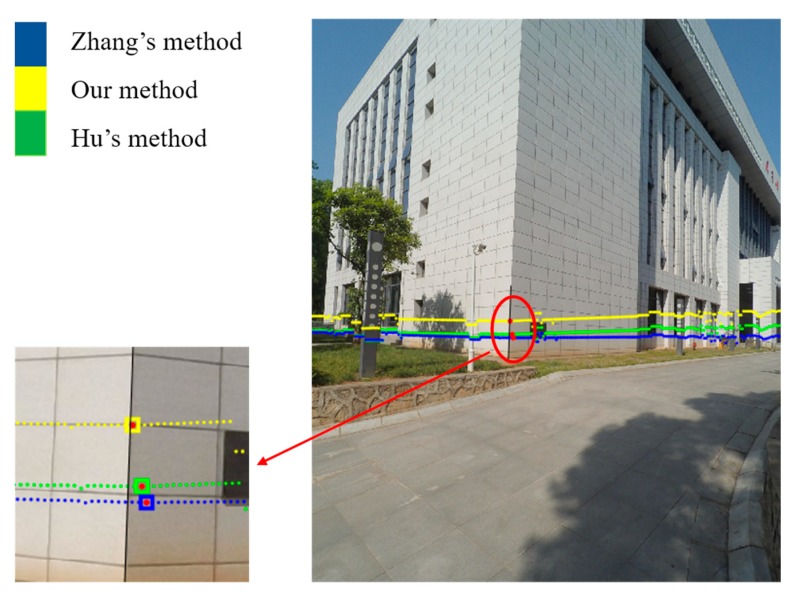
Projection of LRF range data on the image (outdoor).

For the outdoor scene, we collected the image and range data of a building as shown in Figure 17. The integrated sensor was about 9 m away from the target and shot the target obliquely. The projection of the LRF laser points on the image are shown in Figure 17, in which the yellow, blue and green represent the results using our, Zhang’s, and Hu’s extrinsic parameters, respectively. The yellow points on the grey lamppost fit the two edges perfectly, while the blue and green points have a leftward offset referring to the two edges. This finding demonstrates that our calibration result is the most accurate. The linking edge and intersection point are also extracted, as shown in Figure 17. The point-to-edge distance using our, Zhang’s, and Hu’s extrinsic parameters are 3.04, 25.06, and 18.19 pixels, respectively. Our calibration scheme is apparently more accurate. All these experiments verify that our calibration scheme using the photogrammetric calibration control field is more accurate in indoor and outdoor applications whether the target is captured vertically or obliquely.

## 4. Conclusions

With the use of a photogrammetric control field, this paper presents a robust and accurate extrinsic calibration scheme between a camera and a 2D LRF which requires only one shot. Our developed scheme references the two sensors to a common control field to achieve the state-of-the-art performance of extrinsic calibration. First, the room corner of the control field is used to solve the extrinsic parameters of the LRF with respect to the control field. Then, a large number of control points are used to simultaneously calculate the intrinsic and extrinsic parameters of the camera with respect to the control field. Finally, the extrinsic parameters between the camera and the LRF are calculated based on the extrinsic parameters of the camera and the LRF with respect to the photogrammetric control field. The elaborate designed photogrammetric control field provides not only accurate and redundant control points for camera calibration, but also excellent trihedron structure to locate the LRF in the control field. The experimental results verify that the proposed scheme is robust, accurate, and precise, and outperforms the state-of-the-art methods. Experiments on real data verify that the calibration results are effective in indoor and outdoor applications.

We suggest the following directions to further improve and evaluate the accuracy of the proposed scheme in our future research.

1. By placing chessboards or equivalent ones around the control field and capturing images and laser data from different poses, we can obtain more and closed-loop constraints which will improve the accuracy of the extrinsic parameters.
2. We suggest adopting the methods in Reference [29,30] for camera calibration using the photogrammetric control field to meet a higher accuracy requirement.
3. The accuracy and robustness of LRF calibration can be improved using redundant intersections. By elaborately placing multiple trirectangular trihedrons in the control field, the laser scanning plane of the LRF can simultaneously intersect each trirectangular trihedron with three lines, and thus, the redundant intersections are obtained.
4. We recommend moving the integrated sensor on a vehicle or rotation platform to capture precise movement information of the sensor and better evaluate the accuracy of the extrinsic parameters in the object space.

## Figures and Tables

**Table 1:** Mean of the extrinsic calibration errors under different noise levels.

Noise level		Our Method				Hu’s Method				Zhang’s Method			
		*Mean_E_R_* (degree)			*Mean_E_T_* (mm)	*Mean_E_R_* (degree)			*Mean_E_T_* (mm)	*Mean_E_R_* (degree)			*Mean_E_T_* (mm)
Image	Laser	r_1_	r_2_	r_3_	T	r_1_	r_2_	r_3_	T	r_1_	r_2_	r_3_	T
1	1	0.009	0.017	0.019	0.870	0.206	0.082	0.228	15.154	0.299	0.621	0.492	27.927
1	15	0.047	0.249	0.253	12.648	0.216	0.266	0.371	21.589	0.449	0.914	0.720	41.039
1	30	0.096	0.612	0.619	31.110	0.235	0.613	0.684	36.694	0.774	1.334	0.954	54.503
5	1	0.044	0.050	0.058	2.379	1.091	0.389	1.188	80.052	1.365	3.035	2.520	139.077
5	15	0.065	0.255	0.261	12.920	1.030	0.466	1.172	77.240	1.602	3.174	2.472	135.330
5	30	0.101	0.612	0.620	31.004	1.093	0.716	1.398	89.015	1.751	3.464	2.720	151.035
10	1	0.086	0.100	0.114	4.603	2.173	0.786	2.367	158.666	3.385	5.933	4.451	246.641
10	15	0.097	0.284	0.294	14.313	2.157	0.813	2.367	158.466	3.307	6.235	4.815	272.386
10	30	0.128	0.637	0.648	31.908	2.052	0.965	2.362	155.465	3.459	6.625	5.024	286.594

**Table 2:** Standard deviation of the extrinsic calibration errors under different noise levels.

Noise level		Our Method				Hu’s Method				Zhang’s Method			
		*Std_E_R_* (degree)			*Std_E_T_* (mm)	*Std_E_R_* (degree)			*Std_E_T_* (mm)	*Std_E_R_* (degree)			*Std_E_T_* (mm)
Image	Laser	r_1_	r_2_	r_3_	T	r_1_	r_2_	r_3_	T	r_1_	r_2_	r_3_	T
1	1	0.005	0.011	0.011	0.521	0.151	0.053	0.145	9.877	0.199	0.321	0.354	19.859
1	15	0.027	0.184	0.182	9.297	0.157	0.195	0.197	11.089	0.335	0.485	0.505	27.727
1	30	0.057	0.440	0.436	22.316	0.158	0.449	0.425	21.273	0.553	0.667	0.678	39.164
5	1	0.024	0.028	0.030	1.114	0.818	0.247	0.785	54.093	0.957	1.629	1.706	92.758
5	15	0.037	0.181	0.179	9.011	0.748	0.311	0.719	48.512	0.979	1.449	1.650	94.119
5	30	0.060	0.442	0.437	22.119	0.805	0.469	0.753	50.433	1.320	1.788	1.786	97.304
10	1	0.050	0.056	0.062	2.165	1.567	0.497	1.506	102.691	2.315	2.817	2.714	151.223
10	15	0.056	0.188	0.185	9.043	1.591	0.521	1.531	103.585	2.433	3.228	3.200	177.354
10	30	0.074	0.452	0.447	22.193	1.586	0.644	1.517	101.520	2.546	3.143	3.290	185.319
